# Efficient recovery of whole blood RNA - a comparison of commercial RNA extraction protocols for high-throughput applications in wildlife species

**DOI:** 10.1186/1472-6750-12-33

**Published:** 2012-06-27

**Authors:** Doreen Schwochow, Laurel EK Serieys, Robert K Wayne, Olaf Thalmann

**Affiliations:** 1 Department of Ecology and Evolutionary Biology, University of California Los Angeles, 2149 Terasaki Life Science Building, Los Angeles, 90095, USA; 2 Department of Animal Breeding and Genetics, Swedish University of Agricultural Sciences, BMC Husargatan 3, Uppsala, SE75124, Sweden; 3 Division of Genetics and Physiology, Department of Biology, University of Turku, Iltäinen Pitkäkatu 4, Turku, 520014, Finland

**Keywords:** Whole blood, RNA preservation, RNA extraction, DNA contamination, Transcriptome sequencing, Globin transcripts

## Abstract

**Background:**

Since the emergence of next generation sequencing platforms, unprecedented opportunities have arisen in the study of natural vertebrate populations. In particular, insights into the genetic and epigenetic mechanisms of adaptation can be revealed through study of the expression profiles of genes. However, as a pre-requisite to expression profiling, care must be taken in RNA preparation as factors like DNA contamination, RNA integrity or transcript abundance can affect downstream applications. Here, we evaluated five commonly used RNA extraction methods using whole blood sampled under varying conditions from 20 wild carnivores.

**Results:**

Despite the use of minute starting volumes, all methods produced quantifiable RNA extracts (1.4 – 18.4 μg) with varying integrity (RIN 4.6 - 7.7), the latter being significantly affected by the storage and extraction method used. We observed a significant overall effect of the extraction method on DNA contamination. One particular extraction method, the LeukoLOCK™ filter system, yielded high RNA integrity along with low DNA contamination and efficient depletion of hemoglobin transcripts highly abundant in whole blood. In a proof of concept sequencing experiment, we found globin RNA transcripts to occupy up to ¼ of all sequencing reads if libraries were not depleted of hemoglobin prior to sequencing.

**Conclusion:**

By carefully choosing the appropriate RNA extraction method, whole blood can become a valuable source for high-throughput applications like expression arrays or transcriptome sequencing from natural populations. Additionally, candidate genes showing signs of selection could subsequently be genotyped in large population samples using whole blood as a source for RNA without harming individuals from rare or endangered species.

## Background

Adapting to an ever-changing environment is one of the most crucial functions of living organisms. Although we have a profound understanding of how some adaptive mechanisms have shaped phenotypic traits (i.e. [1-3]), their molecular foundation remains largely unexplored in natural populations of vertebrates (i.e. [4,5]). Over the last several decades, molecular studies addressing signatures of selection have mainly focused on model organisms or captive animals for which genome information became readily available and sample material can be easily obtained [6-9]. Even though such studies provide fundamental knowledge for evolutionary biology, similar studies on vertebrates in a natural context have thus far only marginally been explored. With the emergence of high-throughput applications such as expression arrays and next generation sequencing methods (NGS) new opportunities have arisen to investigate the genetic mechanisms facilitating adaptation in natural populations [10-13]. Moreover, the introduction of transcriptome sequencing via RNAseq [14,15] has led to important findings in the field of ecological genomics [16-18]. Owing to their high sensitivity, these novel technologies require special precautions in sample handling and preparation, a pre-requisite not often achievable for free-ranging, natural populations of vertebrates. For example, fresh tissue collection for RNA extraction can be difficult for rare or endangered species. Although sampling via fat or muscle tissue biopsies may be possible in some exceptional cases, the primary least invasive and abundant source of RNA in vertebrates remains whole blood.

Whole blood offers several advantages for transcriptome studies on non-model organisms but is surprisingly underrepresented in the recent literature. Blood has an important role in mediating between the environment and the organism and hence exploring gene expression profiles derived from blood may provide deeper insight into immune response or stress metabolism [19-22]. Furthermore, compared to other tissues, blood is relatively easy to obtain and small volumes can be collected with little harm to the animal. However, extracting useful RNA from whole blood is hampered by several challenges. A first concern is the effective preservation of RNA. RNA is sensitive to endogenous and exogenous RNases, which rapidly degrade the nucleic acid resulting in changes in the expression profile and a potential loss of rare transcripts immediately after sample collection [23-26]. Solutions to this dilemma include immediate snap freezing of the sample in liquid nitrogen or dry ice [27,28], or storage in special whole blood preservation buffers. Snap freezing presents an alternative for some but may be difficult for more remote and unpredictable field situations. In contrast, storage buffers are easy to transport and are purported to increase the time before freezing becomes necessary and thus provide researchers with the flexibility to ship samples from the field.

A second challenge of using whole blood for transcriptome profiling is the heterogeneity of blood cells and the high content of interfering hemoglobin mRNAs. Globin mRNA in mammalian whole blood is estimated to account for 70% of all mRNA transcripts (i.e. [29]), an excess that significantly affects the detection sensitivity of less abundant mRNAs in high-throughput approaches and reduces the amount of target cDNA sequenced [29-32]. In order to minimize the confounding effect of globin mRNA, methods have been suggested which reduce the abundance of those transcripts prior to high-throughput sequencing. These methods range from biotinylated globin capture oligos, to a laborious fractionating of whole blood using microcentrifuges which depletes the primary source of globin mRNA (reticulocytes). Blood samples cannot be frozen or stored in preservation buffer for these approaches prior to processing and hence these methods are impractical for field-based investigations of natural, free-ranging populations. However, a recently launched, commercially available whole blood filter system has been suggested to effectively filter out reticulocytes while capturing and preserving leukocytes, the cell population most interesting for investigations of adaptive response to changing environmental conditions.

As encouraging as these preservation methods appear, to our knowledge, their performance has never been evaluated by concurrent experiments on different species and using similar collection conditions. In the present study, we evaluated four commercially available RNA preservation buffers applied to whole blood samples in seven different carnivore species. Furthermore, we explored the performance and the suitability of a new whole blood filter system for application under field conditions. All five sampling methods were evaluated with regard to RNA quality including DNA contamination and RNA yield as well as the integrity of the isolated RNA. In a proof-of-concept-experiment, we modified and validated conditions of mRNA preparation from whole blood samples for NGS applications. Finally, we provide guidelines on how to preserve and process RNA extracts from whole blood sampled from wildlife populations.

## Results

We evaluated different RNA extraction methods applied to whole blood sampled under field conditions that resemble situations researchers face when investigating non-model organisms in their natural habitat. We used samples collected from 20 carnivores and RNA from each individual was preserved using five methods (four buffers and one filter each; except one individual for which we only collected blood for the four whole blood preservation buffers) resulting in a total of 99 RNA extractions (Additional file 1: Figure S1, Additional file 1: Table S1).

### Presence of contaminants in the RNA extracts from blood samples

DNA might inadvertently be co-extracted with RNA and thus negatively affect downstream applications. Since most commonly used nucleic acid quantification procedures evaluate both RNA and DNA simultaneously, we explicitly tested each RNA extract for potential DNA contamination by means of PCR amplifications.

In total, we examined four PCRs for each extract and found 62 of 80 whole blood non-filtered extracts to contain traces of DNA contamination. When considering bands in the expected sizes, 44.0 – 74.0% of the total PCRs resulted in amplification products (Table 1). The RiboPure™ kit samples showed the highest percentage of amplification and hence the largest amount of DNA contamination. The successive PCRs were distributed among 17 of the 20 tested extracts, leaving only 15.0% free of DNA contamination. Of those RiboPure™ extracts contaminated with DNA, a surprising 30.0% gave products for all four test PCRs. A modest amount of DNA contamination was observed for samples extracted with the PAXgene™ blood kit (overall 54.4% positive PCRs), and lastly, TRIzol® LS and RNeasy® methods gave only 43.8% and 40.0% positive PCRs, respectively.

**Table 1:** RNA integrity, yields and DNA contamination for each tested preservation buffer and extraction method

**Approach**	**Preservation buffer**	**Extraction kit/protocol**	**RIN**	**RNA yield range (μg/ 500 μl blood)**	**260/280**	**260/230**	**DNA contamination**	
							**% Amplification**^**1**^	**% Individuals with min. 1 amplicon**^**2**^
Whole blood	RNA*later*®	RiboPure™	4.6± 2.3	5.0- 43.9	1.9± 0.1	2.1± 0.2	74.0	85.0
	RNAprotect®	RNeasy®	6.9± 2.6	0.0- 6.2	2.1± 0.2	0.9± 0.6	40.0	80.0
	PAXgene™	PAXgene™	7.7± 1.2	0.1- 10.2	2.1± 0.2	1.2± 0.6	54.4	95.0
	TRIzol® LS	TRIzol® LS	6.2± 2.9	0.2- 15.1	1.9± 0.1	1.3± 0.5	43.8	80.0
Filtered blood	RNA*later*®	LeukoLOCK™	7.6± 1.9	0.1- 3.7	2.0± 0.1	0.8± 0.5	11.0	20.0

^1^Percentage of PCRs that resulted in positive amplification. Each PCR performed in four reactions that amplified were counted.

^2^ Percentage of individuals in which a minimum of a single amplification occurred.

We additionally investigated the performance of a commercially available kit (LeukoLOCK™) that filters leukocytes and depletes the amount of reticulocytes prior to extraction. We found that among the 19 pre-filtered extracts only four samples yielded PCR amplifications. This corresponds to only 11% amplification rate (Table 1) and is the lowest contamination rate observed in this study. Despite this low degree of contamination, two positive samples amplified three out of four test products, a result indicating substantial contamination within those affected extracts.

When testing if the extraction method, the species or individual factors of each animal affected the DNA contamination in the 99 RNA samples, only the extraction method revealed a significant correlation (ANOVA: p = 2.948e^-07^; Pearson’s Chi-squared test: p = 1.254e^-09^).

**Figure 1:**
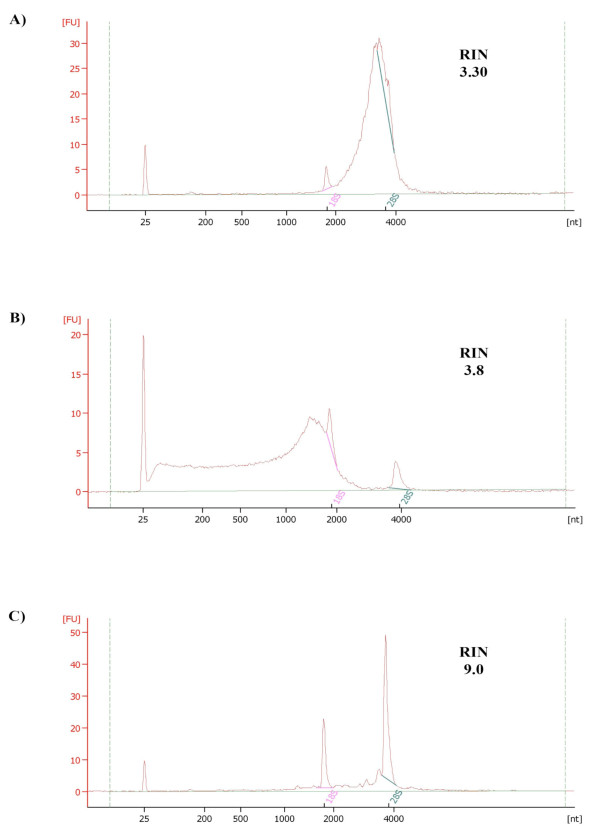
**Electropherograms exemplifying quality differences in various RNA extracts.** Agilent 2100 Bioanalyzer electropherogram obtained from one individual extracted using the Mouse RiboPure™ kit without DNase treatment (**A**); after one DNase treatments (**B**); and using the RNeasy® kit including one DNase treatment (**C**). The broadened peak for the 28 s ribosomal RNA in (**A**) indicates substantial DNA contamination of the RNA samples. After DNase treatment, traces of partially sheared DNA are still visible below the 18 s peak (**B**). Samples extracted using the RNeasy® kit including one DNase treatment, give a clean electropherogram with well-defined 18 s and 28 s peaks with a high RIN value (**C**).

We also detected severe DNA contamination through visual inspection of electropherograms in eight out of the 99 extracts processed on Agilent’s Bioanalyzer (Figure 1). Interestingly, among the eight samples, the majority were extracted using the RiboPure™ method and underscore the tendency of this method to co-extract DNA. Finally, in order to further reduce the DNA contamination of the RNA extracts, we performed a second, more rigorous DNase treatment of all extracts that yielded more than two successful PCR amplifications as well as the eight RNA samples showing visual DNA contamination on the Bioanalyzer. This procedure effectively removed all detectable traces of DNA contaminants.

Besides DNA contamination, the presence of additional contaminants such as proteins, salt and organic compounds were determined by measuring the absorption at wavelengths A260/280 and A260/230 using Nanodrop. We observed significant variation between the different procedures (ANOVA: p = 3.678e^-09^ for A260/280 and p = 6.103e^-08^ for A260/230). The A260/280 ratio averaged between 1.9 and 2.1 for all extraction kits (Table 1), while more variation was observed for A260/230 ratios. Only the RiboPure™ kit resulted in a A260/230 ratio of 2.1 indicating samples free of organic compounds, followed by TRIzol® LS (1.3 ± 0.5), PAXgene™ (1.2 ± 0.6), RNeasy® (0.9 ± 0.6) and LeukoLOCK™ with the lowest ratio (0.8± 0.5).

### RNA yield and integrity

**Figure 2:**
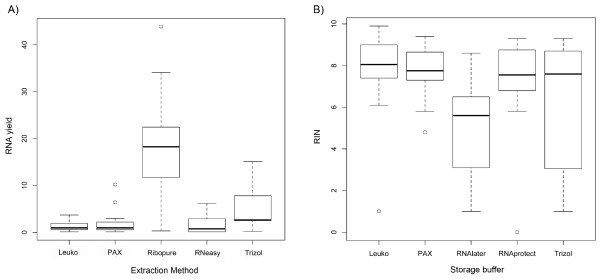
**RNA yield and RIN values.** RNA yield and RIN values for the five different RNA extraction protocols. (**A**) RNA yield (in μg) obtained after one or (if required) two DNase treatments from 500 μl blood using four different protocols/ kits for RNA extraction from whole blood as well as the LeukoLOCK™ filter system. (**B**) RIN values of the RNA extracted from blood samples preserved in different buffers. Please note that samples collected with the RNA filter “Leuko” were stored in RNA*later*® just as the whole blood samples “RNAlater”. The RIN values of both collection methods differ dramatically and represent both the upper and lower limit of the performance of all tested preservation buffers.

After complete depletion of contaminant DNA, we determined the amount of extracted RNA. Starting from minute volumes of only 500 μl whole blood (Additional file 1: Figure S1), we obtained the following RNA yields with significant variation between the applied methods (ANOVA: p = 4.653e^-10^): 5.0-43.9 μg using RiboPure™; 0–6.2 μg for the RNeasy®; 0.1-10.2 μg for the PAXgene™ and 0.2-15.1 μg for samples extracted using TRIzol® LS (Table 1, Figure 2A). The RNA yield range adjusted for 500 μl filtered blood using LeukoLOCK™was amongst the lowest with 0.1- 3.7 μg (Table 1, Figure 2).

We further investigated the integrity of each RNA extract as indicated by the RIN factor and observed the following average values: 4.6 ± 2.3 for samples extracted using RiboPure™ (stored in RNA*later*®); 6.9 ± 2.6 RNeasy® (stored in RNAprotect®); 7.7 ± 1.2 for PAXgene™ (stored in PAXgene™ blood buffer), 6.2 ± 2.9 TRIzol® LS (stored in TRIzol® LS; Table 1, Figure 2B) and lastly a RIN value of 7.6 ± 1.9 for the filtered blood (stored in RNA*later*®). The values for RNA integrity differed significantly between methods (ANOVA: p = 0.007). In comparison to RNeasy® and PAXgene™, whole blood samples extracted with TRIzol® LS or RiboPure™ showed a broad range of well-preserved RNA samples to RNA samples totally degraded. Due to low RNA concentrations in 12 samples, we lowered the threshold parameters in the Agilent Bioanalyzer software for these particular samples in order to obtain a RIN value. These samples included a total of seven study animals (three bobcats, two foxes, one sea lion and one coyote) and all extraction/ storage methods were affected.

We found a trend towards higher average RIN values in extracts collected in a lab-like facility (i.e. in zoo facilities; 7.4 ± 1.4) when compared to those collected in the field (5.8 ± 1.2). Furthermore, we observed a significant effect of the sample collector as well as the species (p = 0.002 and p = 5.872e^-06^, respectively) but these two factors are not mutually exclusive (Additional file 1: Table S1).

### Hemoglobin depletion

**Figure 3:**
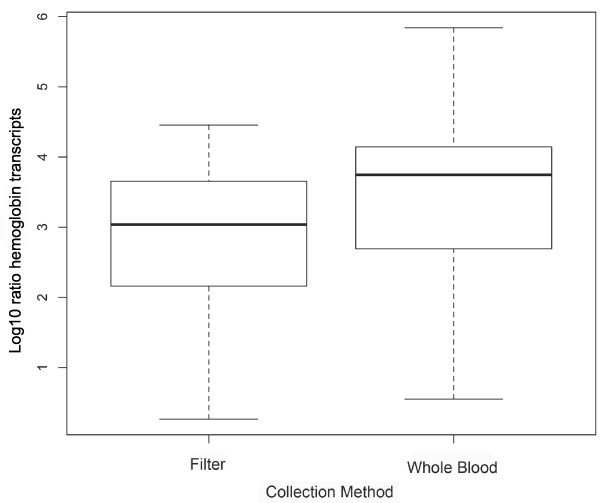
**Log transformed ratio of hemoglobin transcripts.** Log transformed ratio of hemoglobin transcripts abundant in whole blood extracts as well as those derived from the LeukoLOCK™ filter system.

In order to evaluate a potential reduction of hemoglobin transcripts in the filtered extracts, we used a quantitative PCR assay to estimate the relative ratio between hemoglobin and three housekeeping genes and compared them to the values derived from the four other whole blood extraction methods. The average log transformed ratio for hemoglobin in filtered blood was significantly higher than that of whole blood (Figure 3, Student’s t-test, p = 0.005). On average, hemoglobin was reduced by 70% in the extracts processed through the filter. When three outliers are excluded, the average reduction increased to 84%. Interestingly, we found a strong correlation between the log transformed ratios and the species used in this study (ANOVA: p = 4.393e^-16^). However, the species were sampled by different researchers and this could consequently also contribute this association.

### 454 sequencing results

Four cDNA samples were prepared differently for use on a 454 GS FLX Titanium sequencer (Table 2). We first assembled all raw reads per sample. Sample B2 showed the lowest number of reads (17,047) with 4,391 reads (25.76%) assembled into 309 contigs. Sample A2 had the largest number of reads (38,601). Fifty percent of those reads (19,318) were assembled into 2,286 contigs. The remaining two samples A1 and B1, yielded 30,145 and 21,219 reads, from which 30.31% and 61.62% built 1,066 and 275 contigs, respectively. Interestingly, the number of singletons (reads only occurring once and not assembled in any contig), did not correlate with the total number of reads per sample. For example, the sample with the lowest number of reads (B2) yielded the highest number of singletons (65.51%), the second highest percentage of singletons (63.71%) was found in sample A1, which in turn showed the second largest number of total reads (Table 2). We detected 36.47% and 28.22% singletons in samples A2 and B1, respectively. This result suggests that the number of total reads does not necessarily determine the abundance of singletons, but additional factors such as tissue type or stochastic effects need to be considered.

**Table 2:** Sample preparation procedure and 454 sequencing results

**Sample ID**	**Preparation steps**	**# raw reads**	**# reads in contigs**	**# contigs**	**# singletons**	**% globin reads**
A1	ribo-minus > 1^st^ & 2^nd^ strand cDNA synthesis > **NORM** > 2^nd^ amp	30,145	9,138	1,066	19,205	5.46
A2	wholeRNA > 1^st^&2^nd^ strand cDNA synthesis > 2^nd^ amp > **NORM** > 3^rd^ amp	38,601	19,318	2,286	14,079	1.95
B1	globin-minus > ribo-minus > 1^st^&2^nd^ strand cDNA synthesis	21,219	13,075	275	5,987	24.10
B2	globin-minus > ribo-minus > 1^st^&2^nd^ strand cDNAsynthesis > **NORM** > 2^nd^ amp	17,047	4,391	309	11,168	0.35

# counted.

Abbreviations used for preparation steps are explained in the Supplementary Information Additional files.

We investigated the effectiveness of the different whole blood sample preparation methods to exclude hemoglobin transcripts. We extracted the genomic regions harboring hemoglobin genes from the publicly available dog genome [33] and mapped all raw reads of each sample to this hemoglobin reference. Although we used various methods to deplete globin transcripts from each sample, sequences mapping to the reference region could be found in every sample (Table 2). A surprising 24.06% of all reads obtained from sample B1 mapped to the reference region, followed by 5.46% of the reads in sample A1 and 1.95% of sample A2. The least number of reads mapping to the globin reference was found in sample B2 (0.35%), suggesting that the preparation method applied to this sample most effectively depleted globin transcripts.

Lastly, we were interested in the number and identity of genes that are present in each of the four samples. The two samples with the largest numbers of contigs, A2 and A1 also yielded the largest numbers of assigned genes, 486 and 292 respectively. However, a higher percentage of assigned contigs were observed in samples B1 with 57.0% (157 out of 275 contigs) and B2 with 35.0% (109 out of 309 contigs). We extracted the number of reads per gene in order to evaluate a potential over-representation of particular genes in the sample. Additional file 1: Table S2 summarizes the top 10 genes with the largest number of reads per sample and in concordance with the results described above, *globin* genes can be found in three samples, indicating its omnipresence.

## Discussion

### Evaluation of RNA preservation buffers and extraction kits for whole blood

The principle aim of this study was to assess the performance of commercially available kits and protocols commonly used to preserve and extract RNA from blood samples. We used total RNA yield, RNA preservation (RIN) and the degree of DNA contamination as measures of performance.

DNA contamination is particularly worrisome if expression profiles are generated with high-throughput methods because even a single contaminating molecule can be detected, making sequencing efforts less efficient and potentially leading to false biological conclusions. Our DNA contamination tests did reveal a substantial amount of DNA co-extracted with all RNA extraction protocols for whole blood. Between 80.0-95.0% of the whole blood RNA extracts resulted in at least one single DNA amplicon (Table 1). We observed significant differences in the extent of contamination depending on the extraction method used, which might reflect the efficacy of the different techniques to remove DNA. For instance, the RiboPure™ protocol is based on a guanidinium thiocyante- phenol- chloroform homogenate that is extracted under low pH. This procedure is known to avoid co-extraction of DNA [34] and therefore the observed 85.0% contaminated extracts were quite unexpected. Such heavy contamination might be the result of carryover DNA from the interface during the phenol-chloroform extraction used in this procedure.

The applied DNase depletion protocols recommended for each of the tested method should in theory have removed moderate amounts of DNA (<50 μg DNA/ ml RNA extract). Surprisingly, irrespective of the extraction method, an additional DNase treatment was required in order to ultimately deplete any trace of DNA, which indicates severely contaminated RNA extracts. This is somewhat alarming and in agreement with results reported for other RNA extraction methods [35]. Our finding implies that great care should be taken in order to generate endogenous RNA sequence reads on any of the NGS platforms.

Aside from DNA, other contaminants of RNA extracts derive from incomplete removal of cellular components such as proteins, lipids and carbohydrates or traces of salt and organic solvents stemming from the extraction procedure itself. With regard to protein depletion in RNA extracts, determined by the A260/280 ratio, all methods yielded samples with a ratio averaging above 1.9, which is considered suitable for NGS [36,37]. In contrast, when considering other organic substances (i.e. Phenol) and aromatic compounds (i.e. Trizol) that absorb at 230 nm wavelength, only the RiboPure™ kit yielded satisfying results with a A260/230 ratio above 1.8, a threshold indicating a low level of contamination. Although some studies have reported reduced efficiency of sensitive, downstream applications due to such contaminants [36] subsequent sample processing might eliminate these contaminants and the actual effect could be negligible when compared to that caused by DNA contamination.

After depletion of interfering DNA, an accurate quantification of extracted RNA was possible. Depending on the extraction kit, the RNA yield for 500 μl blood is supposed to range between 1.6 μg up to 55 μg. While the yield obtained for PAXgene™ samples is similar to that suggested by the manufacturers (0.1-10.2 μg/500 μl blood vs. 1.6- 3.2 μg/500 μl blood as tested by the manufacturer), the amount of RNA retrieved using the RNeasy® kit was much lower than stated (0–6.2 μg/500 μl blood vs. 25–35 μg/ 500 μl blood). Many factors, such as the individual hydration condition of an animal or blood density differences in certain parts of the body [34] can influence the amount of RNA obtained from blood samples and help to explain the observed variation.

Intriguingly, our study showed that samples extracted using organic solvents like phenol/chloroform (e.g. RiboPure™ kit) or TRIzol® LS reagent resulted in the highest RNA yields (see also [36,38], Figure 2A). While the combination of phenol/chloroform and RNA bound to glass fiber columns (as employed by the RiboPure™ kit) seemed to be very efficient in retrieving RNA [36], the purely column-based approaches not incorporating organic solvents (PAXgene™ and RNeasy®) yielded about five times lower average RNA quantities.

We observed a significant effect of the collector on the RNA yield (ANOVA: p = 0.02) as well as the preservation of the RNA (ANOVA: p = 0.002). The reasons for this pattern might include individual differences in sample handling procedures. However, we need to stress the fact that the effect of collectors and species could not be differentiated since in most cases samples from one species were collected by a single person under unique conditions (Additional file 1: Table S1). Consequently, our results imply that either single or correlated effects may potentially cause differences in RNA yield.

Aside from retrieving sufficient RNA quantities, its integrity is yet another, perhaps even more important factor affecting downstream applications [37]. If not stored properly, low abundance and long transcripts are rapidly degraded by tissue specific RNases. This process can affect RNA integrity and result in changes of the biological expression profile [23-26]. The PAXgene™ (samples stored in PAXgene™ blood buffer) and RNeasy® kits (samples stored in RNAprotect®) both yielded extracts with the highest RIN values, which makes them most suitable for sensitive downstream applications. These two methods also produced the extracts with the narrowest range of RNA preservation variation and are among the easiest to use, which highlights the suitability of these methods whenever samples are collected under difficult, non-laboratory conditions.

Notably, the two methods resulting in the highest yield of RNA (TRIzol® LS, and RiboPure™ with samples stored in RNA*later*®) produced the extracts with the lowest and most widely scattered RIN values rendering its suitability for high-throughput applications questionable. This was surprising since both storage buffers have extensively been used to preserve RNA from a broad range of animal tissues including brain, liver, testis, tissue culture cells or white blood cells in many different species (e.g. [9,39-41]). The TRIzol® LS reagent used herein is a ready-to-use solution for isolation of total RNA from liquid samples of different origins (TRIzol® LS). One possible explanation for the weak performance of this method might be the unconventional application of the storage buffer under field conditions. More precisely, the solution was aliquoted into RNase free tubes and provided to collectors. The time elapsed until actual usage of the buffer for sample collection ranged from a few weeks to two months and we observed a decrease in volume in some of these tubes, probably due to evaporation. Furthermore, it is recommended to keep the TRIzol® LS solution away from higher ambient temperatures and light, factors often unmanageable in field sites and shown to affect DNA recovery from fecal samples [42]. In fact, we found a significant difference between the average RIN values of TRIzol® LS samples collected under field conditions (RIN = 4.4) and in zoo-like facilities (RIN = 7.9; p = 0.005). The inefficiency of TRIzol® to serve as preservation agent was previously also reported by Chiari and Galtier [34]. In summary, apart from its toxicity, our findings suggest that TRIzol® LS would not be the method of choice for blood collection in the field, especially in warmer climates.

Another commonly used RNA preservation solvent for whole blood samples is RNA*later*® (e.g. [9]). We found a surprisingly weak performance of this preservation method. According to the manufacture’s information high amount of proteins present in whole blood samples might lead to insoluble precipitation with RNA*later*®. One observation supporting this possibility was the high amount of coagulated blood sampled in RNA*later*®, a pattern not prevalent in any of the other preservation buffers storing the same blood volume from the same animals. Since the preservation process of RNA*later*® relies upon the permeability of the tissue cells, it is possible that large blood precipitates hamper the chemical’s ability to pass through the cell membrane and interact with the RNA. This is in accordance with other studies reporting that the size of solid tissue material stored in RNA*later*® negatively affects the RNA integrity [36,37]. However, whenever RNA*later*® was used in conjunction with the filter system it served as the best preservative for RNA as we discuss below.

### General performance of a filtering method and its effect on hemoglobin reduction

We tested the performance of another RNA extraction method that *a priori* filters leukocytes from whole blood and by depleting an excess of hemoglobin mRNA, it ultimately reduces the amount of downstream inhibitors in RNA extracts. However, this filtering procedure comes with a cost as the average yield of 1.2 μg/ 500 μl blood in the LeukoLOCK™ filter system was the lowest in our analysis. Although low in quantity, the RNA itself was well preserved as indicated by high RIN values (Table 1, Figure 2B). Only four out of 19 samples showed a RIN below 7, and we argue that these rare cases might be due to inappropriate handling of the filter system by collectors in the field. The handling process itself is more elaborate and requires a certain degree of laboratory skill and precautions. It is a multi-step procedure, which is cumbersome in the field, particularly if researchers aim to preserve the RNA immediately after blood collection. In field settings that require animal handling, researchers are often more focused on the animal itself and are shorthanded so the immediate processing of blood through the filter post collection may be difficult.

An encouraging aspect of this particular system was the low amount of DNA contamination observed in all RNA extracts. Only four of 19 samples yielded positive test PCRs but only two of them amplified in more than three PCRs. The actual co-extraction of DNA from filtered blood might be lower due to the efficient removal of the majority of erythrocytes. Whereas, mature mammalian erythrocytes contain neither a nucleus nor cell organelles like mitochondria, immature reticulocytes released from the bone marrow still harbor a nucleus. These cells constitute about 1% of the total number of circulating erythrocytes, which themselves number between four and six million in a single μl of human blood. This leaves about 40,000 – 60,000 nuclei exclusively derived from immature red blood cells and given the sensitivity of PCRs (i.e. [43]), this number is sufficient to result in positive test PCRs whenever these cells are not depleted *a priori*.

Insufficient elimination of reticulocytes from whole blood RNA extracts not only affects DNA contamination but might also change the ratio of highly abundant versus rare transcripts. Hemoglobin is highly expressed in blood and warrants special treatment [29,32,44]. A system such as LeukoLOCK™ results in the removal of 70% of hemoglobin transcripts. However, other extraction methods require additional treatments to deplete these transcripts which may be necessary to allow the detection of potential candidates of selection such as low abundant transcripts of disease related genes [29,45]. Methods for hemoglobin reduction are readily available but in most cases are designed exclusively for model organisms.

### Efficiency of RNA preparation methods for NGS

Whether finding the causative mutations or simply cataloguing the wealth of expressed genes is a priority, normalizing the RNA extract is beneficial [46,47]. In agreement with previous studies, we found that normalized samples produced the highest number of contigs but also singleton reads, which are equivalent to rare transcripts that might harbor valuable information otherwise not accessible [16,48]. Normalization of RNA extracts effectively reduces highly abundant transcripts from the blood but in cases such as the omnipresent hemoglobin, additional measures need to be considered. Various methods depleting hemoglobin prior to sequencing exist and can be readily applied at different stages of sample preparation. We show that the application of such methods in combination with normalization contributed to a substantial reduction of reads representing hemoglobin when compared to the 25.0% hemoglobin reads apparent in the non-normalized sample (B1). This effect of normalization is only beneficial in qualitative assessments of an organism’s transcriptome but should be avoided whenever quantification of gene expression is desired.

## Conclusions

The ultimate decision about which RNA extraction method to apply may reflect a balance between performance, the anticipated laboratory effort, the project’s logistics and finances and the scope of the project. Considering all tests employed in this study, we conclude that the most promising RNA storage and extraction method is the LeukoLOCK™ filter system. Besides its performance, the method includes practical amenities well suited for field applications. First, the RNA*later*® soaked membrane of the filter system allows flexibility in handling time as the RNA is stable for 24 hours at 37 °C, one week at 25 °C and one month or more at 4 °C. Second, the LeukoLOCK™ system works reliably even with small amounts of starting material. The system was originally designed for 9 ml human blood but we have demonstrated that much smaller volumes (1 ml whole blood) are sufficient as well and might therefore become the method of choice for small species or individuals that are difficult to handle or suffering from stress. One caveat about the system is the complex sample processing, which requires a certain familiarity with the steps in the protocol. However with appropriate practice and precautions the benefits of this system outweigh the extra effort, particularly in remote areas with limited access to electricity and refrigeration.

The second best performing method was the PAXgene™ kit. The preservation buffer is intended for stabilization of blood for as much as three days at 18- 25 °C or for 5 days at 2-8 °C. This method has been designed for medical applications and is therefore more expensive than other kits. We would like to stress that great care has to be taken to ensure a sufficient removal of DNA co-extracted with this RNA extraction kit.

We show that whole blood can be a valuable resource for transcriptome studies and that the efficiency of NGS technologies can be maximized by employing appropriate library preparation methods (i.e. normalization). Consequently, whole blood is a promising and easy accessible RNA source that has great potential for NGS studies in ecological and conservation genetics.

## Methods

### Sample collection for RNA preservation and extraction evaluation

Whole blood samples were collected from seven carnivore species, including four Mexican wolves (*Canis lupus baileyi*), four grey foxes (*Urocyon cinereoargenteus*), three bobcats (*Lynx rufus*), two coyotes (*Canis latrans*), four California sea lions (*Zalophus californianus*), two harbor seals (*Phoca vitulina*) and one California sea otter (*Enhydra lutris*) in different facilities/ field settings (Additional file 1: Table S1). In order to stabilize and preserve the RNA, we first collected 3 ml whole blood from each individual into an EDTA coated blood tube. Using a sterile syringe, approximately 0.5 ml of whole blood was transferred into each of four tubes containing unique RNA preservation buffers. These buffers were: RNA*later*® (Ambion), RNAprotect® (Qiagen), PAXgene™ blood buffer (PreAnalytiX, Qiagen) and TRIzol® LS Reagent (Invitrogen) (Additional file 1: Figure S1). Whereas RNA*later*® and RNAprotect® buffers and tubes were used as provided by the manufacturer, 1500 μl of TRIzol® LS Reagent was aliquoted into RNase free 2 ml tubes assuring a blood to buffer volume ratio of 1:3. A similar volume adjustment was performed according to Carrol et al. (2007) using the PAXgene™ blood buffer [49]. The remaining 1 ml whole blood was processed directly from the EDTA tube through a pre-assembled LeukoLOCK™ filter system (Ambion). The filter was flushed with 2 ml PBS (Ambion) and subsequently with 2 ml RNA*later*®. Once the filter was saturated with RNA*later*®, it was sealed until further use. All samples were shipped to the lab at UCLA immediately after collection in the field and kept at −20 °C until further processing. Each individual’s blood was sampled at the same time for all five approaches simultaneously, except for one coyote where we failed to obtain a LeukoLOCK™ sample (see Additional file 1: Table S1). The samples were collected according to national guidelines for handling animals and each institution has acquired the respective permits required for sampling wild animals. All procedures were performed by highly qualified personnel and followed strict ethical guidelines.

### Laboratory methods

#### RNA extraction and quality assessment

The samples were extracted in three batches using commercially available kits by following manufacturer’s protocols (Table 1). However, some modifications were incorporated when using the PAXgene™ buffer as suggested for low amounts of starting materials [49]. Species and order of samples were randomized between batches as well as during extractions.

Extracted RNA samples were subjected to kit specific or recommended DNase treatments. The efficiency of DNA removal was determined in “no-RT” control PCR using a regular polymerase. We targeted four loci (see section below) by using the following conditions. A mastermix was prepared with 0.2 U TaqGold (Applied Biosystems), 1x buffer Gold, 1.5 mM MgCl_2_, 4% DMSO, 0.2 mM each dNTP, 0.5 μM forward and reverse primer mix and 1 μl of the non-transcribed RNA. The PCR was performed on an Eppendorf Mastercycler EP Thermal cycler (Eppendorf) using the following cycling steps: initial denaturation at 94 °C for 9 minutes, denaturation at 94 °C for 20 seconds, annealing temperature 56 °C for 20 seconds, extension at 72 °C for 20 seconds; final extension at 72 °C for 4 minutes; 40 cycles total. PCR amplification was evaluated on a 2% Agarose gel stained with SYBR Safe DNA gel stain (Invitrogen) and visualized under UV light. Samples that successfully amplified in at least two out of the four PCRs were subjected to an additional DNase treatment using 4 U of rDNaseI (Ambion) according to the manufacturer’s recommendation.

In order to evaluate the RNA yield, 10 μl of 1:10 diluted, DNA-free RNA was used in a Quant-iT™RiboGreen® RNA reagent assay (Invitrogen), which uses a dye that intercalates with nucleic acids and absorbance (260 nm) could be measured at with a multimode DTX880 plate reader (Beckman Coulter). RNA integrity was assessed at the UCLA Sequencing and Genotyping Core using the RIN values calculated on an Agilent 2100 Bioanalyzer (Agilent Technologies).

#### cDNA synthesis and target gene amplification

In order to assess whether prior filtering (LeukoLOCK™ filter system) would efficiently reduce the amount of hemoglobin transcripts compared to the whole blood approaches, we performed a qPCR targeting ß- hemoglobin as well as three housekeeping genes for all extracted samples. A volume of 4 μl undiluted DNA-free RNA was reverse transcribed using SuperScript III First-Strand Synthesis Super Mix (Invitrogen). Since random hexamers have shown to result in an overestimation of mRNA copy number by up to 19-fold [50], we used oligo(dT) primers instead.

Primers were designed based on a multi species alignment and are available upon request. Previous findings in humans [51] suggest that three expressed housekeeping genes are sufficient to normalize qPCR data from leukocytes. Here we used Ubiquitin C (*Ubc*), Phospholipase A2 (*YWHAZ*) and the human acidic ribosomal protein (*HuPo*; [52]).

1^st^ strand cDNAs were diluted 1:100. Additionally, samples that were filtered through the LeukoLOCK™ system were first diluted 1:2 in order to account for the 1 ml whole blood starting material. 1 μl of each dilution was used in a 10 μl reaction of LightCycler 480 SYBR Green I Master mix (Roche). All samples from one individual were run at the same time in duplicates and on the same plate to reduce nuisance parameters such as technical variations within individuals. We included two DNA samples as a positive control/ positive calibrator on each plate. All primers were used in a 0.5 μM end concentration. The LightCycler protocol included 5 minutes activation at 95 °C, 10 seconds denaturing at 95 °C, primer annealing for 15 seconds at 55 °C, elongation for 30 seconds at 72 °C and a melting curve (95 °C for 5 sec, 65 °C for 2 min and 97 °C). The last step was a cooling step at 40 °C for 10 sec. The samples were subjected to 50 cycles. In addition to the qPCR set up, we evaluated the PCR efficiency for each primer pair by including 12 diluted DNA samples (original concentration, 1:2, 1:10 and 1:100).

#### Sample preparation for 454 sequencing

Blood samples from two confined wolves (Mexican and Arctic wolf) were collected into PAXgene™ blood tubes and prepared for sequencing utilizing Roches’ 454 GS FLX Titanium platform following four different procedures. After the extraction of RNA, each extract was treated differently (Table 2), allowing us to compare the performance of different methods such as globin reduction or enzymatic normalization. The actual 454 GS FLX Titanium library preparation steps included blunt ending of small DNA fragments, 454 GS FLX Titanium adapter ligation, various purification steps, emulsion PCR and a final release of single-stranded DNA fragments. A more detailed description is provided in the Supplementary Information. As a proof of concept, we sequenced each prepared library on a single 1/16 lane of a 454 GS FLX Titanium run.

### Data analyses

#### DNA contamination, RNA yield and integrity, and hemoglobin reduction

To first evaluate the extent of DNA contamination of each RNA extract, we used two different analytical approaches. Assuming that higher concentration of DNA in an extract is more likely to yield amplification success, we first counted the number of positive PCR’s for each extraction method and performed a Pearson’s Chi square test. We further calculated the ratio of positive PCRs for each sample and performed an ANOVA using the statistical software package *R* (http://www.R-project.org).

Total RNA yield was estimated using the Quant-iT™RiboGreen® RNA reagent assay comparing the estimates of RNA concentrations against a provided standard and adjusted for a starting volume of 500 μl whole blood. To obtain a reliable and comparable measure for the integrity of all extracted RNA samples, we used the RNA integrity number (RIN), a measurement that uses the entire electrophoretic trace of each RNA sample. We used the Agilent 2100 Bioanalyzer and accompanied software which calculates the RIN values within a range of 0 and 10, where 0 represents a totally degraded and 10 a completely preserved RNA sample [53]. An ANOVA was performed in order to find correlations between the extraction methods and RNA yield, the storage buffer and RIN factor as well as other variables such as collection conditions.

To assess the amount of hemoglobin transcripts for the whole blood and the filtered approach, we processed the outputs from the LightCycler runs (using LightCycler 480 software) by first undertaking a melting curve analysis in order to determine that each PCR worked properly and no unspecific amplification occurred [54]. Samples that didn’t result in clean melting curve patterns (i.e. double peaks indicating nonspecific amplification) were omitted from further analysis and the qPCR was repeated. In the next step, we estimated the relative ratios between our target (*ß- hemoglobin*) and the reference genes (*HuPoUbcYWHAZ*) using the relative quantification software (Efficiency-Method, Applied Bioscience). This approach compensates for differences in target and reference gene amplification efficiency within an experiment and between experiments. The PCR efficiency for each primer pair was evaluated once and used as an external standard throughout the experiment. The target was paired with all three reference genes and the geometric mean of the resulting ratios was calculated. By equalizing the average ratios of the whole blood approaches with 100% of globin transcripts, we were able to determine the average reduction of *ß- hemoglobin* in the filtered samples. To evaluate the significance of *ß- hemoglobin* reduction in filtered compared to whole blood samples, we performed both an ANOVA as well as a Students t-test.

#### Sequence assembly and 454 data analysis

After on-machine filtering of raw reads, all retained reads of each sample were assembled into contigs using Roche’s gsAssembler software with minimum overlap of 40 bp and minimum overlap identity of 90%. In order to evaluate each sample treatment (Table 2) for usefulness in preparing a transcriptome library from blood samples, the data was furthermore parsed for total number of contigs, reads per contig and total number of singletons.

In order to assess the number of reads derived from hemoglobin transcripts, we extracted the genomic regions containing hemoglobin genes from the publicly available dog genome [33] (chr.21:31,293,338-31,325,315 and chrUN:46,802,419-46,803,081) and used this as a mapping reference for Roche’s gsMapper software applying the following filters: a minimum overlap of 40 bp and 95% minimum identity. Lastly, to evaluate the number of annotated genes sequenced for each of the four preparation methods, we used the program Blast2GO [55] to annotate all resulting contigs to known genes.

## Competing interest

The authors declare that they have no competing interest.

## Authors' contributions

DS and OT developed the idea, designed the study and performed the experiments and the analysis. LEKS provided the majority of the blood samples and RKW contributed to the study design as well as provided funding. DS and OT wrote the manuscript with editing contributions from all authors. All authors read and approved the final manuscript.

## Supplementary Material

Additional file 1 **Supplementary Information.** The accompanied pdf-file provides in depth information regarding the sample preparation for 454 sequencing, two additional tables and a figure that depicts the sampling set up [56-58]. Click here for file
