# Supplementary material for: Efficient recovery of whole blood RNA - a comparison of commercial RNA extraction protocols for high-throughput applications in wildlife species
Source: BMC Biotechnol. 2012 Jun 27;12:33. doi: 10.1186/1472-6750-12-33 (PMC3406948; doi:10.1186/1472-6750-12-33)
Supplement: Additional file 1 — Supplementary Information. The accompanied pdf-file provides in depth information regarding the sample preparation for 454 sequencing, two additional tables and a figure that depicts the sampling set up [56-58]. [file 1472-6750-12-33-S1.pdf]

Supplementary information

RNA preparation for 454 sequencing

2.5 ml whole blood from two confined wolves (Californian Wolf Center) were collected into original PAXgene™ blood tubes (PreAnalytiX, Qiagen) and immediately after collection shipped on ice to our lab where they were kept at -20 ºC until further extraction.

The extraction was performed following the protocol for whole blood samples collected in PAXgene™ blood tubes (including DNase treatment). The concentration and purity of the samples was measured using a Nanodrop (Thermo Scientific) and an aliquot of each sample was run on an Agilent 2100 Bioanalyzer to assess RNA integrity (UCLA Sequencing and Genotyping Core). The samples yielded 20.2 µg and 10.2 µg for the Mexican and Arctic wolf respectively with a RIN value above 7.

While one sample (Arctic wolf, sample A in Table 2) was not subjected to hemoglobin depletion, the other sample (Mexican wolf; sample B in Table 2) underwent hemoglobin removal ("globin-minus" in Table 2) according to manufacturer's protocol (GLOBINclear™ Mouse/Rat, Ambion). This procedure resulted in five aliquots, which were subsequently ethanol-precipitated as followed: all samples were pooled and ammonium acetate was added to a final concentration of 2.5 M. Pure ethanol (vol 2.5) was added, mixed and the sample incubated for 50 min at -20 °C. The precipitated RNA was pelleted by centrifugation for 15 min at 12,000 rpm (4 °C). The supernatant was removed and the sample washed with 500 µl 70% ice-cold ethanol followed by another centrifugation step (5 min, 12,000 rpm, 4 °C). The washing step was repeated once. Afterwards the sample was air dried for 5 min at room temperature and resuspended in RNase free water by pipetting up and down.

The precipitated samples, were split into two aliquots (A1/A2 and B1/B2, respectively). Both aliquots of the Mexican wolf (samples B1 and B2) underwent removal of ribosomal RNA ("ribo-minus", Table 2) as well as one sub-sample of the Arctic wolf (samples A1). We used the RiboMinus™ Transcriptome Isolation Kit (Human/ Mouse; Invitrogen) according to the manufacturers' recommendations.

Three µl RNA of each wolf sample were reverse-transcribed following the manufacturers' protocol (Mint cDNA synthesis Kit, Evrogen). We determined the optimal numbers of cycles of the 2nd strand synthesis for each sample individually. This is required because PCR overcycling of 1st strand cDNA yields nonspecific PCR products whereas too few cycles result in low yields. Both are undesirable for high-throughput applications such as next generation sequencing applications. After the evaluative PCR, the 1st strand cDNA obtained from the total RNA sub-sample of the Artic wolf (sample A2) was further amplified with 18 additional cycles on the thermal cycler whereas all remaining sub-samples were subjected to additional 17 PCR cycles.

To remove remaining primer excess, buffers and the enzymes from 1st strand cDNA products, the cDNA was run on a 0.8% low melting point agarose gel and extracted using Zymoclean Gel DNA recovery Kit (Zymo Research). During this process we also removed fragments of 750 bp length for all samples, potentially representing hemoglobin transcripts. A total of three purified extracts (A1, B1 and B2, ", Table 2) were used for normalization of the cDNA (Trimmer Kit, Evrogen). The normalization of full-length-enriched cDNA libraries (**NORM,** Table 2) is based upon the selective ability of the enzyme duplex-specific nuclease (DSN) to preferably cut double stranded DNA. Very abundant transcripts will be degraded until only an equalized single-stranded cDNA fraction will remain intact [1, 2]. This procedure is supposed to allow a more sufficient detection of low abundant transcripts. The ¼ dilution of DSN provided the best results for all experimental samples. After normalization all three samples were subjected to 13 PCR cycles for 1st amplification. The full-length-cDNA libraries were purified using the gel extraction procedure as described above followed by 12 PCR cycles for a second amplification. The second amplification step became necessary to increase the proportion of long transcripts in the cDNA sample. The process of PCR tends to favor the amplification of smaller products over larger ones, which can result in the loss of rare, long transcripts. Using specific primers provided with the kit we were able to ensure that longer transcripts were amplified more effectively than smaller ones.

Sequencing library preparation and 454 sequencing

Novel pyrosequencing techniques require specific library preparation steps in order to facilitate the fragment size suitable for the respective method, the ligation of adapters and an accurate quantification of the copy number prevalent in the final library. We started the 454 GS FLX Titanium library preparation from the gel-extracted cDNAs and skipped the nebulization step as visual inspection of the agarose gel revealed only a small proportion of fragments being longer than 1,000 bp. Since we were interested in the full spectra of mRNA transcripts, we also did not remove small fragments from the cDNA pool. The samples were further subjected to blunt ending, 454 Titanium adapter ligation, various purification steps and a final release of single-stranded DNA fragments from the capture beads. This resulted in 30 µl of single stranded DNA library and to quantify the yield we used a procedure suggested in [3] which relies upon the efficiency of quantitative PCR. As suggested [3], we established a DNA standard using an old Titanium library, which was amplified using the 454 GS FLX Titanium emPCR priming sites as annealing targets and evaluated the copy number via the respective DNA concentration in the purified PCR product. The PCR product was diluted to cover a range from E^{02} to E^{09} copies. All samples and the standard dilutions were set up for a qPCR on Roche's LightCycler 480 (Roche, Indianapolis) using the company's High Resolution Meltcurve kit. In addition to the kit reagents we added 2 µm final concentration of each emPCR equivalent primer to the qPCR set up and run the following protocol: 10 min pre-incubation at 95°C followed by 45 amplification cycles each consisting of 10 sec denaturing at 94°C, 15 sec primer annealing at 60°C and 45 sec elongation at 72°C. The quantification occurred during each of the 45 elongation steps. An approximate of 2 x E^{06} total copies of each sample was subsequently processed in 454 sequencing set up following to the manufacturers' instructions. Each sample was individually sequenced on a 1/16th lane of a full 454 sequencing run.

References

1. Zhulidov PA, Bogdanova EA, Shcheglov AS, Shagina IA, Wagner LL, Khazpekov GL, Kozhemyako VV, Lukyanov SA, Shagin DA: **A method for the preparation of normalized cDNA libraries enriched with full-length sequences**. *Russian Journal of Bioorganic Chemistry* 2005, **31**(2):170-177.
2. Zhulidov PA, Bogdanova EA, Shcheglov AS, Vagner LL, Khaspekov GL, Kozhemyako VB, Matz MV, Meleshkevitch E, Moroz LL, Lukyanov SA *et al*: **Simple cDNA normalization using kamchatka crab duplex-specific nuclease**. *Nucleic Acids Research* 2004, **32**(3):e37.
3. Meyer M, Briggs AW, Maricic T, Höber B, Höffner B, Krause J, Weihmann A, Pääbo S, Hofreiter M: **From micrograms to picograms: quantitative PCR reduces the material demands of high-throughput sequencing**. *Nucleic Acids Research* 2008, **36**(1):e5.

Supplementary Table1. Species replicates and condition for samples used.

Species	Replicates	Facility/ Area	Condition	Collector
Mexican wolf (*Canis lupus baileyi*)	4	Californian Wolf Center	Lab-like**	1
Grey fox (*Urocyon cinereoargenteus*)	4	Santa Monica Mountains	Field	2
Bobcat (*Lynx rufus*)	3	Santa Monica Mountains	Field	2
Coyote (*Canis latrans*)	2*	Different Nature Reserves in Orange County	Field	3
Harbor seal (*Phoca vitulina*)	2	Aquarium of the Pacific	Lab-like**	4
California sea lion (*Zalophus californianus*)	4	SeaWorld San Diego	Lab-like**	5
California sea otter (*Enhydra lutris*)	1	Aquarium of the Pacific	Lab-like**	4
**Total**	**20**			

* Blood from one of the individuals has only been collected for the whole blood and not for the LeukoLOCK™ approach.
** Samples have been collected in a zoo-like facility or sanctuary.

Supplementary Table 2. Top 10 assigned genes with most abundant reads per sample.

A1 Gene match	A1 % Assigned reads	A2 Gene match	A2 % Assigned reads	B1 Gene match	B1 % Assigned reads	B2 Gene match	B2 % Assigned reads
PREDICTED: hypothetical protein [Bos taurus]	12.5	5-aminoevulinate synthase 2	7.7	protein	11.2	unknown [Zea mays]	13.1
beta globin	9.4	line-1 reverse transcriptase homolog	5.6	delta- synthase 2	8.9	rRNA promoter binding protein	7.3
hemoglobin subunit delta	8.2	PREDICTED: hypothetical protein LOC100425210 [Macaca mulatta]	3.5	rRNA promoter binding protein	8.3	PREDICTED: hypothetical protein [Bos taurus]	7.0
af095770_1pth-responsive osteosarcoma d1 protein	6.8	protein	3.2	af095770_1pth-responsive osteosarcoma d1 protein	6.8	chk1 checkpoint homolog (pombe)	5.5
hypothetical protein TcasGA2_TC002334 [Tribolium castaneum]	5.4	hba_canla ame	2.6	chk1 checkpoint-like protein	4.8	unnamed protein product [Tetraodon nigroviridis]	4.8
senescence-associated protein	4.7	beta globin	2.5	hba_canla ame	4.1	af095770_1pth-responsive osteosarcoma d1 protein	4.4
cytoplasmic 2	2.2	unnamed protein product [Tetraodon nigroviridis]	1.4	PREDICTED: hypothetical protein [Gallus gallus]	3.3	senescence-associated protein	3.9
PREDICTED: hypothetical protein, partial [Monodelphis domestica]	2.1	chk1 checkpoint homolog (pombe)	1.2	s100 calcium binding protein a8	2.9	5-aminoevulinate synthase 2	3.5
PREDICTED: similar to predicted protein [Equus caballus]	2.0	cationic amino acid transporter 3-like	1.2	phosphoribosyl transferase domain containing 1	2.7	desmocollin 2-like	3.5
notch homolog 2	2.0	kelch-like 25	1.1	PREDICTED: hypothetical protein LOC100471656 [Ailuropoda melanoleuca]	2.6	PREDICTED: hypothetical protein, partial [Monodelphis domestica]	3.5

Supplementary Figure 1: Schematic diagram depicting the procedure applied to each sample included in the extraction protocol comparison

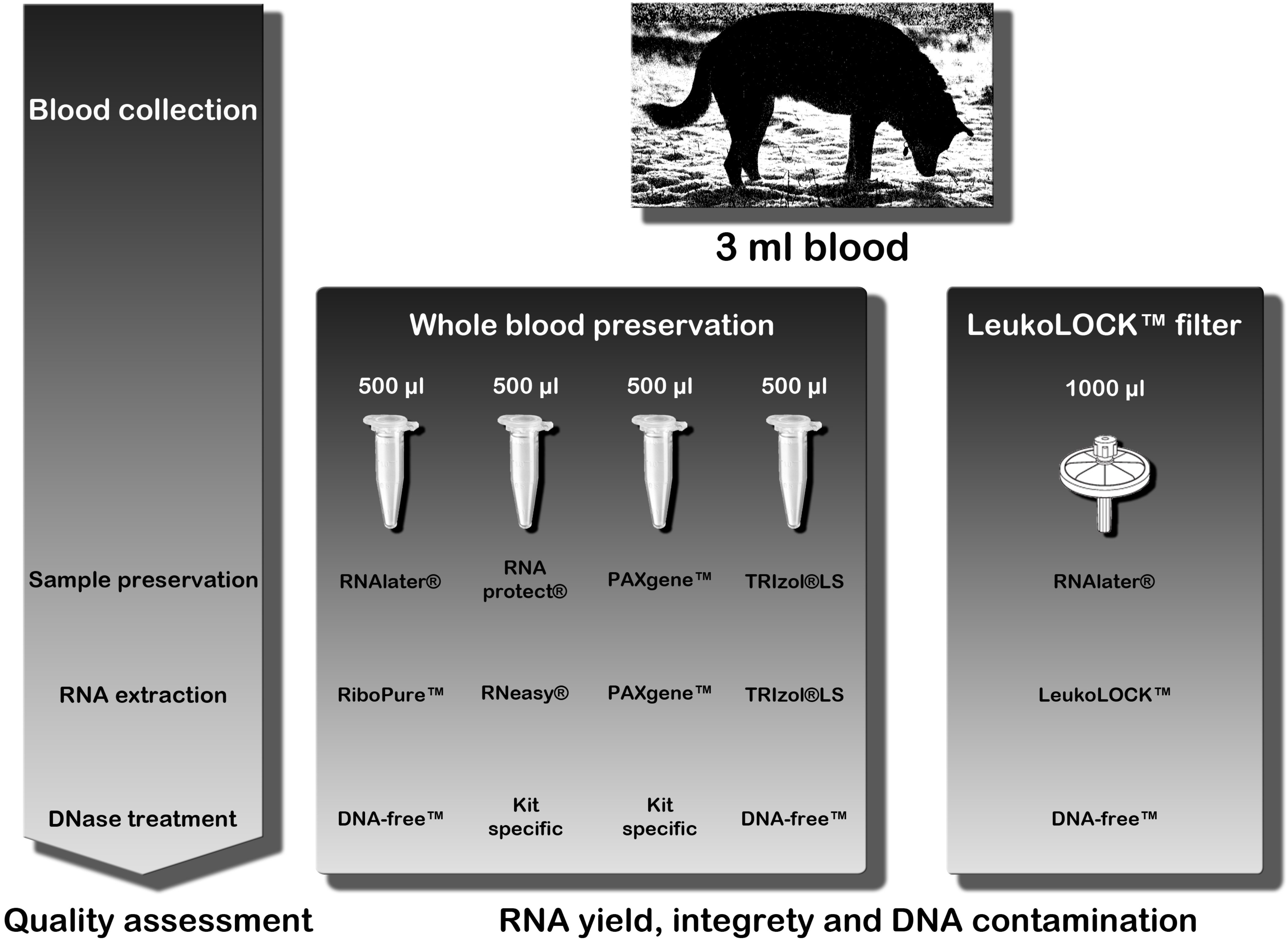
Blood collection
3 ml blood
Whole blood preservation
LeukoLOCK™ filter
500 µl
500 µl
500 µl
500 µl
1000 µl
Sample preservation
RNAlater®
RNA protect®
PAXgene™
TRIzol®LS
RNAlater®
RNA extraction
RiboPure™
RNeasy®
PAXgene™
TRIzol®LS
LeukoLOCK™
DNase treatment
DNA-free™
Kit specific
Kit specific
DNA-free™
DNA-free™
Quality assessment
RNA yield, integrety and DNA contamination
